# C. DIFFICILE INFECTION AS A MAKER FOR DYSBIOSIS IS LONGITUDINALLY ASSOCIATED WITH COGNITIVE DECLINE AND DEMENTIA

**DOI:** 10.1093/geroni/igac059.000

**Published:** 2022-12-20

**Authors:** Nicholas Resciniti

**Affiliations:** University of Southern California, Los Angeles, California, United States

## Abstract

Disruption of the gut microbiome (dysbiosis) is associated with cognitive decline and dementia; however, there is a lack of findings demonstrating how dysbiosis is longitudinally associated with cognitive outcomes. This study aimed to quantitatively assess whether there is an association between Clostridium difficile infection (CDI) as a marker for dysbiosis and cognitive decline and dementia. We used 6,147 older adults from the Health and Retirement Study and the Centers for Medicare and Medicaid Services from 1999-2012. Outcome variables included cognitive scores (0-27) and dementia (8 or less). The exposure was time-varying ICD-9 CDI. Generalized estimating equation were used for the main analysis. Having at least one CDI infection was associated with -2.85 (B = -2.85; 95% CI: -4.43, -0.26) at 2000 (baseline) and at 2002 (B = -2.85; 95% CI: -4.43, -0.26), 2004 (B = -2.85; 95% CI: -4.43, -0.26), 2006 (B = -2.85; 95% CI: -4.43, -0.26), and 2008 (B = -2.85; 95% CI: -4.43, -0.26), after adjusting. Those having at least one CDI had 6.62 greater odds (OR = 6.62; 95% CI: 1.68, 26.05) of dementia at 2000 and was associated with time points 2002 (OR = 4.12; 95% CI: 1.48, 11.52) and 2004 (OR = 2.57; 95% CI: 1.14, 5.81). This study indicated that those with a CDI had a greater cognitive decline and a greater probability dementia. These findings provide evidence that dysbiosis has a larger impact on cognitive outcomes closest to the initial disruption and is less impactful further on.

